# Transthyretin cardiac amyloidosis

**DOI:** 10.1093/cvr/cvac119

**Published:** 2022-08-05

**Authors:** Aldostefano Porcari, Marianna Fontana, Julian D Gillmore

**Affiliations:** National Amyloidosis Centre, Division of Medicine, University College London, Royal Free Campus, Rowland Hill Street, London NW3 2PF, UK; Center for Diagnosis and Treatment of Cardiomyopathies, Cardiovascular Department, Azienda Sanitaria Universitaria Giuliano-Isontina (ASUGI), University of Trieste, Trieste 34149, Italy; National Amyloidosis Centre, Division of Medicine, University College London, Royal Free Campus, Rowland Hill Street, London NW3 2PF, UK; National Amyloidosis Centre, Division of Medicine, University College London, Royal Free Campus, Rowland Hill Street, London NW3 2PF, UK

**Keywords:** Transthyretin cardiac amyloidosis, Cardiac magnetic resonance, Cardiac scintigraphy with bone tracers, Prognostic stratification, Therapy, TTR

## Abstract

Transthyretin cardiac amyloidosis (ATTR-CA) is an increasingly recognized cause of heart failure (HF) and mortality worldwide. Advances in non-invasive diagnosis, coupled with the development of effective treatments, have shifted ATTR-CA from a rare and untreatable disease to a relatively prevalent condition that clinicians should consider on a daily basis. Amyloid fibril formation results from age-related failure of homoeostatic mechanisms in wild-type ATTR (ATTRwt) amyloidosis (non-hereditary form) or destabilizing mutations in variant ATTR (ATTRv) amyloidosis (hereditary form). Longitudinal large-scale studies in the United States suggest an incidence of cardiac amyloidosis in the contemporary era of 17 per 100 000, which has increased from a previous estimate of 0.5 per 100 000, which was almost certainly due to misdiagnosis and underestimated. The presence and degree of cardiac involvement is the leading cause of mortality both in ATTRwt and ATTRv amyloidosis, and can be identified in up to 15% of patients hospitalized for HF with preserved ejection fraction. Associated features, such as carpal tunnel syndrome, can preceed by several years the development of symptomatic HF and may serve as early disease markers. Echocardiography and cardiac magnetic resonance raise suspicion of disease and might offer markers of treatment response at a myocardial level, such as extracellular volume quantification. Radionuclide scintigraphy with ‘bone’ tracers coupled with biochemical tests may differentiate ATTR from light chain amyloidosis. Therapies able to slow or halt ATTR-CA progression and increase survival are now available. In this evolving scenario, early disease recognition is paramount to derive the greatest benefit from treatment.

## Graphical Abstract

**Graphical Abstract:**
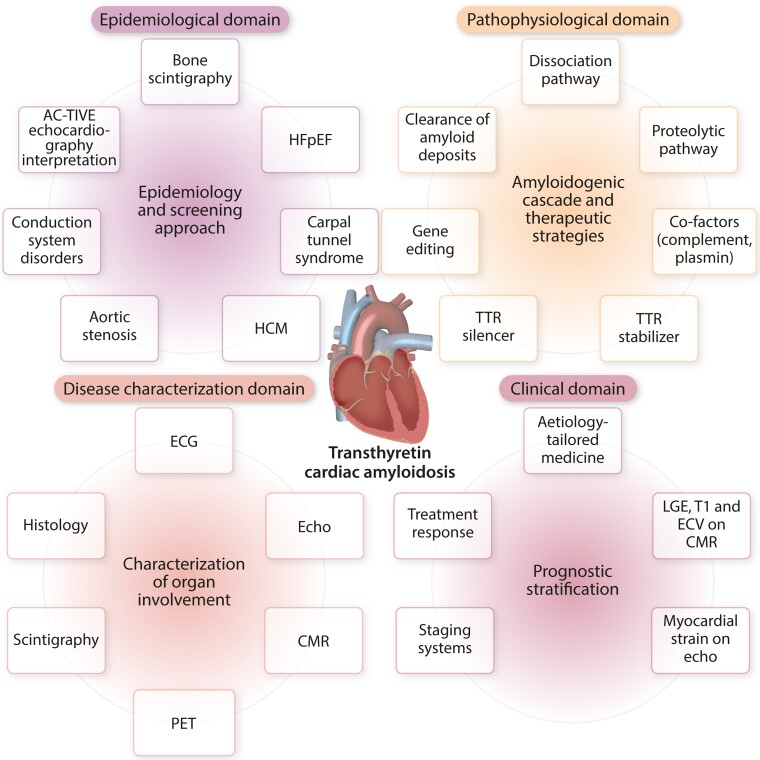
**Grey zones and future directions in transthyretin cardiac amyloidosis.** AC-TIVE, interpretation of echocardiographic findings according to the AC-TIVE study; ATTR, transthyretin amyloidosis; CMR; cardiac magnetic resonance; ECG, electrocardiography; ECV, extracellular volume; HCM, hypertrophic cardiomyopathy; HFpEF, heart failure with preserved ejection fraction; LGE, late gadolinium enhancement; PET, positron emission tomography; SPECT; single-photon emission computed tomography; TTR, transthyretin.

**This article is part of the Spotlight Issue on Heart Failure**

## 1. Introduction

Systemic amyloidosis encompasses a heterogeneous family of diseases induced by deposition of misfolded proteins in the form of amyloid fibrils within the extracellular space of various organs.^1^ More than 30 precursor proteins are known to misfold and self-assemble with highly ordered abnormal cross β-sheet conformation forming insoluble fibrillary material with a relatively stable core structure, which is resistant to proteolysis.^1,2^ Amyloid deposits are histologically identifiable by characteristic apple-green birefringence when stained with Congo-Red dye and examined under cross-polarized light.^1^ Immunohistochemical staining of amyloid with a panel of antibodies against specific precursor proteins is the most widely available technique for characterization of amyloid fibril type, but inconclusive results occur in up to 30% of cases.^3^ As confirmation of the amyloid fibril type is essential to direct clinical management and disease-modifying therapy, in such inconclusive cases, the use of immunogold electron microscopy and mass spectrometry confer the greatest sensitivity and specificity for amyloid typing.^2,3^

Transthyretin (TTR) amyloidosis, also called ATTR amyloidosis, is an underappreciated, life-threatening disease characterized by progressive deposition of misfolded or cleaved TTR protein in organs.^1,2^ This condition results from age-related failure of homoeostatic mechanisms in wild-type ATTR (ATTRwt) amyloidosis (non-hereditary form) or destabilizing mutations in variant ATTR (ATTRv) amyloidosis (hereditary form).^4^ Disease occurs when aggregation of amyloid fibrils in the extracellular space disrupts the structure, integrity and function of the affected tissue. In clinical practice, ATTRwt amyloidosis manifests as a predominant cardiomyopathy [transthyretin cardiac amyloidosis (ATTR-CA)], while ATTRv amyloidosis is typically associated with polyneuropathy (ATTR-PN) as well as cardiomyopathy.^5^

Prognosis is dependent on age at disease onset, time from disease onset to diagnosis (i.e. diagnostic delay), amyloidogenic mutation (in ATTRv amyloidosis) and phenotype (cardiac and/or neurologic). Although amyloidosis is a multi-system disease, the presence and severity of cardiac involvement is the leading predictor of mortality both in ATTRwt and ATTRv amyloidosis.^2,4^ Amyloid deposition in the heart leads to expansion of the extracellular space with associated disruption in myocardial architecture, systolic and diastolic function.^6^ The increase in myocardial mass determines a progressively smaller ventricular cavity size, resulting in fixed end-diastolic volume. ATTR-CA is slowly progressive and clinically well tolerated until marked ventricular wall thickening, severe diastolic dysfunction and conduction system disease have occurred.^1,2^

Traditionally, the diagnosis of ATTR-CA was established only by endomyocardial biopsy (EMB), predominantly in late stages of disease, with a median time interval of 4 years from the onset of cardiac symptoms.^7^ The recognition of ATTR-CA has increased exponentially over the last few years. Major advances in imaging such as scintigraphy with bone tracer and cardiac magnetic resonance (CMR) have heralded a non-invasive approach to diagnosis of ATTR-CA, which can now be achieved without recourse to histological demonstration of amyloid in ≈70% of cases.^8^ Furthermore, landmark developments in therapies that inhibit hepatic synthesis of TTR, stabilize the tetramer, or disrupt fibrils have been reported to slow or halt disease progression in ATTR-PN and to improve survival in ATTR-CA.^9^

Advances in non-invasive diagnosis, coupled with the development of effective treatments, have shifted ATTR-CA from a rare and untreatable disease to a relatively prevalent condition that clinicians should consider on a daily basis.^2^

This review will address the epidemiology, pathogenesis, diagnosis, and treatment of ATTR-CA related to wild type and variant TTR forms and provide insights into future perspectives in the field.

## 2. Pathogenesis

**Figure 1:**
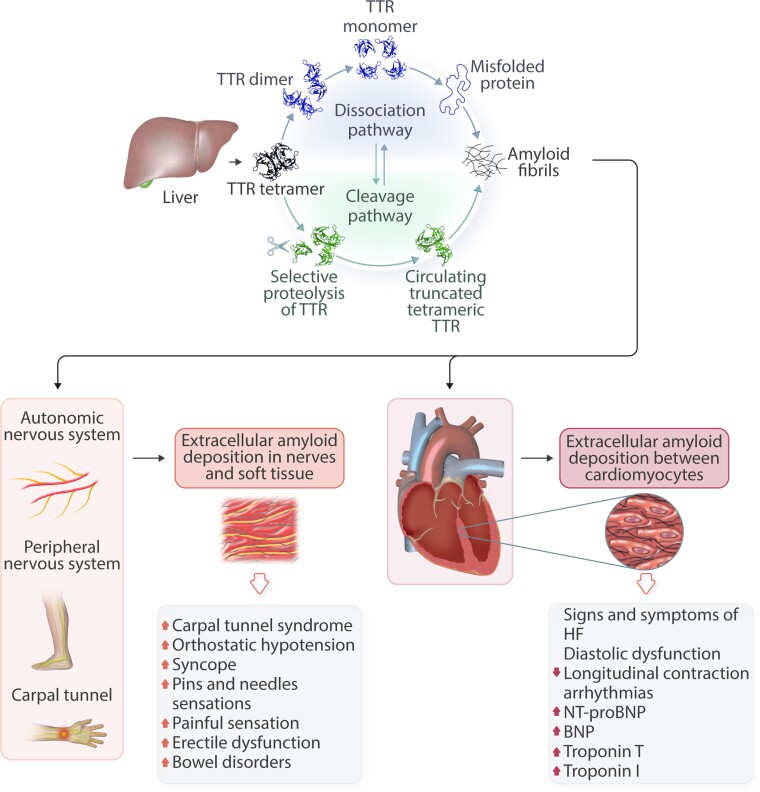
Pathophysiology of transthyretin synthesis with main pathways of the amyloidogenic cascade and consequences of organ involvement. BNP, brain natriuretic peptide; HF, heart failure; NT-proBNP, N terminal brain natriuretic peptide; TTR, transthyretin.

TTR, formerly named pre-albumin, is a highly conserved 55-kDa protein composed of four monomers that circulates as a homo-tetramer and functions as a carrier protein for thyroxine and retinol binding protein (RBP).^10^ The native TTR protein is primarily synthesized in the liver and secreted into the blood, with lesser amounts produced by the choroid plexus and retinal pigmented epithelial cells **(***Figure 1*). TTR has a native globular structure with a ligand-binding hydrophobic channel at the centre of the tetramer, between the dimers.^10^ TTR monomers are rich in beta strands and exhibit an intrinsic propensity to aggregate into amyloid fibrils. Besides its functions as a carrier protein, TTR has a proteolytic activity involved in the cleavage of apoA-I, neuropeptide Y and Aβ peptide.^10^

The amyloidogenic cascade is a complex process, not completely elucidated, and several mechanisms are involved. In vitro, the conversion of TTR into amyloid fibrils starts when the tetrameric form of TTR becomes unstable and the protein dissociates into dimers and monomers that misfold into a non-native conformation. TTR dissociation is the crucial and rate-limiting step in amyloidogenesis under laboratory conditions. Factors that appear to promote TTR instability and shift the equilibrium towards the monomer state include oxidative modifications, age-related failure of cellular homeostatic mechanisms, metal cations and genetic mutations.^11^ Misfolded monomers self-assemble in soluble, non-fibrillar oligomers, presumably amyloid fibril precursors, with significant cytotoxic effects on tissues and, later, aggregate and accumulate as amyloid deposits. Beside TTR tetramer dissociation, a proteolytic pathway for amyloid formation has recently been elucidated using the Ser52Pro TTR variant. This single amino acid substitution promotes susceptibility of the TTR tetramer to selective proteolytic cleavage, resulting in the release of the C-terminal residue 49–127 fragment, which is potently amyloidogenic particularly under conditions of shear stress.^12^ This finding strongly suggests that proteolytic cleavage of the native tetrameric TTR variant with formation of the residue 49–127 polypeptide is a distinct pathway of TTR amyloidogenesis in vivo.^12^

These events lead to the formation of a nucleus with enough stability to grow by monomer addition. This stochastic process occurs under particular conditions (i.e. concentration, temperature) in the so-called lag or nucleation phase. Notably, the addition of preformed seeds can significantly shorten or even complete this phase (seeding phenomenon).^13^ Later, in the elongation phase, the addition of monomers to the nucleus results in formation of amyloid fibrils. These fibrils can be composed of a mixture of both C terminal fragments and full-length TTR (type A fibrils) or by full-length TTR only (type B fibrils).^14^ The extremely slow rate of progression (i.e. years) of this process in vitro suggests that catalysing factors are involved in vivo.^13^

The hallmark of ATTR amyloidosis is the extracellular deposition of aggregated TTR or TTR fibrils in tissues. Whether these deposits directly induce organ dysfunction or represent epiphenomena is debated. Data support a central role of oligomers as causative agents of amyloid-associated cellular dysfunction.^4,13^ Circulating oligomeric species, rather than mature amyloid fibrils, seem to participate in the mechanism of tissue damage with important differences in ATTR compared to light chain (AL) amyloidosis. In AL amyloidosis, circulating species have been demonstrated to exert direct ‘toxic’ effects on cell membranes with activation of apoptotic pathways and disruption of tissue architecture and function.^15^ Conversely, the main mechanism of damage in ATTR amyloidosis is related to progressive amyloid deposition in the organ, while the toxic effect of small TTR oligomeric species is less relevant. These differences in pathophysiological mechanism explain the clinical observation of larger amount of amyloid burden in the heart with relatively preserved ejection fraction in ATTR-CA compared to smaller amyloid burden with severe systolic dysfunction in AL-CA.^4^

## 3. Epidemiology and clinical phenotype

Historically, amyloidosis has been considered a rare condition affecting fewer than 0.5 people in 100 000 and is listed among rare diseases within international rare diseases’ databases such as Orphanet (www.orpha.net) and National Organization for Rare Disorders (https://rarediseases.org). Over the years, advances in cardiac scintigraphy with bone tracers for non-biopsy confirmation of ATTR-CA and CMR imaging, both of which yield highly characteristic findings in ATTR-CA, have revolutionized the diagnosis of this condition.^4^ The development of treatments increasing survival promoted population screening campaigns aimed at reaching an early diagnosis.

Contemporary knowledge of the epidemiology of ATTR-CA relies mostly on real-world studies using in- or outpatient claims data, or registries of diagnosed patients.^16^ Recent data from the Medicare beneficiaries in the United States from 2000 to 2012 showed that, in hospitalized patients over 65 years of age, the incidence rate of CA (both AL and ATTR amyloidosis) was 17 per 100 000 person-years and the prevalence rate was 55 per 100 000 person-years.^16^ In Sweden, the prevalence of diagnosed ATTR-CA increased steadily from 1.0 per 100 000 in 2008 to 5.0 per 100 000 in 2018.^16^ Data from the Danish national registry showed an increase in the incidence of CA over the last two decades from 0.88 to 3.56 per 100 000 person-years in the general population ≥65 years old with a median diagnostic delay from overt heart disease to CA diagnosis of approximately 1 year.^16^ The increase in the median age at diagnosis and the frequency of male patients suggested that ATTRwt amyloidosis was driving this increase. Data from the National Amyloidosis Centre (NAC) in London, which is centrally commissioned as the single centre in the United Kingdom for diagnosis and monitoring of amyloidosis, suggested that AL amyloidosis remained the most common type, accounting for 55% of all cases in the period 1987–2019, and the diagnosis of ATTRwt-CA has increased exponentially from an incidence of less than 3% in the period 1987–2009 to 25% between 2016 and 2020.^17^ Nevertheless, there are still significant delays in establishing the diagnosis of ATTR-CA following presentation with cardiac symptoms, up to >4 years in >40% patients with ATTRwt-CA.^7^ Although the epidemiological figure is still under scrutiny, ATTR-CA is more common than traditionally thought and is currently recognized as an emerging cause of HF and mortality worldwide.

The Transthyretin Amyloidosis Outcomes Survey (THAOS) registry is the largest ongoing, worldwide, longitudinal, observational survey of patients presenting with ATTR amyloidosis. The THAOS registry confirmed that ATTR amyloidosis is a heterogeneous condition with significant regional differences in demographic characteristics, distinct mutations, and clinical manifestations (*Table 1*). The majority (> 70%) of patients in the United States are older men with ATTR-CA due to wild-type or Val122Ile TTR mutation compared to the rest of the world.^5^ Data from this registry provided information on natural history and outcomes in ATTR amyloidosis and demonstrated the presence of three main phenotypes at presentation: cardiomyopathy (ATTR-CA), polyneuropathy (ATTR-PN) and mixed. The presence of ATTR-CA, either in isolation or in combination with polyneuropathy, was found in one-third of subjects and conferred poor survival.

**Table 1:** Characteristics of wild-type and common variant ATTR amyloidosis

TTR type	Area	Prevalence	Male/female ratio	Median age of onset	Affected organs
Wild-type	Worldwide	> 25%^a^	25:1 to 50:1	>70–75 y	Heart, Soft tissue
Val122Ile	United States, Caribbean, Africa	3–4%^b^ Afro-Caribbean	3:1	> 70 y	Heart, PNS, Soft tissue
Val30Met	Portugal, Sweden, Japan	1:1000^b^	2:1	> 50 y (late-onset) 30 y (early-onset)	Heart, PNS/ANS
Thr60Ala	United Kingdom, Ireland	1%^b^ Northwest Ireland	2:1	> 45 y	Heart, PNS/ANS

ANS, autonomic nervous system; PNS, peripheral nervous system; TTR, transthyretin; y, years.

Prevalence histologically rather than clinically.

Prevalence of the mutation.

In ATTR amyloidosis, patients presenting with a cardiac phenotype commonly have signs and symptoms of HF, atrial fibrillation (AF), increased LV wall thickness and normal LVEF. Extracardiac involvement in ATTRwt amyloidosis is commonly related to the presence of carpal tunnel syndrome (CTS), which is often the earliest presenting symptom and may precede cardiac symptoms by several years.^1,2,18^ Polyneuropathy is more commonly associated with ATTRv amyloidosis. ATTR-PN is predominantly axonal involving both the small and large fibres and presents as a combination of progressive sensory motor peripheral neuropathy and autonomic neuropathy.^5^ It commonly starts with loss of the small fibre-mediated sensations of heat or cold, sometimes associated with pain. Autonomic neuropathy causes a number of manifestations such as erectile dysfunction, postural hypotension, early satiety, and diarrhoea or constipation (or both).^5^ Of note, neuropathy can be subtle and should be actively sought even in patients with TTR variants more commonly associated with dominant cardiomyopathy, up to 50% of whom may have neurological manifestations.^19^ ATTR amyloidosis is associated with nonspecific symptoms and a high index of suspicion is paramount in achieving the correct diagnosis.

## 4. Wild type transthyretin amyloidosis

ATTRwt-CA predominantly affects males and presents later on in life. This condition has mostly been recorded as an incidental finding at post-mortem examination in the heart of 25–40% of unselected adults >80 years and in 32% of patients >75 years with heart failure with preserved ejection fraction (HFpEF).^4,6^ Following the validation of non-invasive diagnostic criteria, screening studies in high-risk populations showed a greater prevalence of occult ATTR-CA than previously expected, indicating that the burden of disease is underestimated. The prevalence of ATTR-CA in patients with HFpEF has been investigated in several studies with heterogeneous inclusion criteria.^16^ The largest analysis on 286 patients ≥60 years with HF and LVEF ≥40% reported a prevalence of disease of 6.3%. Gonzalez-Lopez *et al.*^20^ found a disease prevalence of 13% among 120 patients ≥60 years hospitalized for HFpEF and LV wall thickness ≥12 mm.^16^ Lindmark *et al.*^21^ reported a frequency of 15% in 86 patients with HFpEF and LV wall thickness >14 mm.^16^ In a histological study on EMB, Hahn *et al.*^22^ found a disease prevalence of 14% in 108 patients with HFpEF due to unclear aetiology.^16^ Among patients with CTS, the prevalence of ATTR-CA ranged from 14% in patients undergoing bilateral CTS surgery, aged ≥60 years, with LV thickening, and without occupational risk factors, to 33% in men with LV thickening and bilateral CTS surgery.^16^ ATTR-CA was diagnosed in 9% of patients with an initial diagnosis of hypertrophic cardiomyopathy (HCM).^16^ Among patients with severe aortic stenosis referred to surgery or percutaneous replacement, the average prevalence of ATTR-CA was 10%, ranging from 4 to 16%, based on inclusion criteria and diagnostic pathway.^16^ Recently, in a large-scale Italian national survey including 5315 consecutive unselected echocardiograms of patients ≥55 years, the prevalence of ATTR-CA was 1%, raising to 23.5% in patients with LV wall thickening and LVEF ≥ 50% with at least one echocardiographic red flag of infiltrative disease.^23^

The heterogeneity in epidemiological estimates of ATTR-CA is related to the different clinical scenarios, inclusion criteria and adopted diagnostic approaches. Nevertheless, all these studies investigating the prevalence of ATTR-CA have contributed to rewrite the epidemiology of the disease and demonstrate that this condition is relatively common in several settings when it is actively screened for.

## 5. Hereditary transthyretin amyloidosis

The TTR gene is found on chromosome 18 and more than 130 pathogenic mutations have been described, resulting in a variable phenotypic presentation, ranging from selective cardiac involvement (ATTR-CA), to pure polyneuropathy with autonomic dysfunction (ATTR-PN) and to mixed phenotype.^24^ Typically, variant TTR is induced by a single-nucleotide substitution resulting in missense mutations and is commonly inherited in an autosomal dominant fashion with variable penetrance.^7^ There is a strong association between the specific mutation, clinical phenotype and prognosis with an overall 4-year survival of 16, 40 and 79% in valine to isoleucine at position 122 (Val122Ile), threonine to alanine at position 60 (Thr60Ala) and valine to methionine at position 30 (Val30Met) mutations.^7^ Of note, carrying a TTR variant is not always associated with future development of disease as poorly characterized epigenetic and non-genetic factors (i.e. age, gender, geographical distribution and endemic/nonendemic aggregation) are involved.^4^ For example, complement activation has been demonstrated to co-localize with amyloid deposits in amyloidotic neuropathy.^25^ In a transgenic C1q deficient mouse model of ATTR Val30Met amyloid neuropathy a 60% increase in amyloid deposition was observed, but elucidation of the role of complement in amyloidogenesis is under investigation.^25^

There is great degree of variability, both in the age of onset as well as penetrance among different populations.^5,26^ The presence of common founders—the ‘founder effect’—is the reason why many mutations tend to cluster in certain ethnic groups and in limited geographical areas owing to migration across Europe and United States: Val30Met is endemic in Portugal (mostly early onset) and Sweden (mostly late onset), while Val122Ile was predominantly observed in France and United States, probably due to the higher proportion of African descendants in these populations.^5^

Val30Met TTR variant is the most common disease-causing TTR variant in Europe and is associated with the development of early-onset (< 50 years) ATTRv-PN or late-onset (> 50 years) ATTRv-CA variably associated with neuropathy.^26^ In the THAOS registry, the majority of Val30Met subjects in Portugal were early-onset Val30Met, while the majority in Sweden were late-onset Val30Met.^26^ Among the TTR variants that predominantly target the heart, Val122Ile is the most common pathogenic TTR variant in the United States and is carried by 3–4% of the Afro-Caribbean and African American population.^5^ Disease penetrance is estimated to be around 20%, accounting for 25 000 affected individuals in the United States.^5^ Val122Ile TTR variant is associated with late-onset (median age of 69 years) severe cardiac involvement with highly symptomatic HF, commonly, with New York Heart Association (NYHA) class > II, lower 6-min walk times, and higher NT-proBNP levels and increased rates of incident HF compared to other TTR variants.^27^ Patients carrying this TTR mutation have increased mortality rates and the shortest median survival from diagnosis among all ATTRv subgroups.^27^ In this population, early diagnosis of ATTRv-CA is challenged by the higher frequency of hypertension and increased LV wall thickness.^27^ Thr60Ala is one of the most common causes of ATTRv amyloidosis in the United Kingdom, being carried by 1% of the population in northwest Ireland.^28^ Median delay from symptom onset to diagnosis is around 2 years and patients predominantly have a mixed cardiomyopathy and neuropathy phenotype.^28^ When ATTR-CA is diagnosed, genetic testing to identify mutations in *TTR* should be performed in all patients, given the important implications for family members and the potential for genetic counselling.^2,4,26^

## 6. Clinical presentation of ATTR amyloidosis

### 6.1 Clinical phenotype

ATTR-CA is often misdiagnosed with a median of 17 hospital attendances per patient to reach the correct diagnosis.^7^ The heart is the main organ involved in ATTRwt amyloidosis and can be selectively or predominantly involved in combination with nerves in ATTRv amyloidosis.^5^ Clinical presentation is highly variable, especially in ATTRv amyloidosis, when considering the worldwide disease profile.^26^

While ATTRwt-CA is mostly found in elderly men, ATTRv-CA presents at a younger age and has greater variability in gender predominance based on the specific TTR variant.^5,26^ Patients with ATTR-CA most commonly present with signs and symptoms of HF and increased LV wall thickness with impaired diastolic filling and longitudinal systolic function.^2,7^ Although ATTR-CA has traditionally been considered the paradigm of restrictive cardiomyopathy, an increasing number of patients are currently diagnosed in earlier disease stages in recent years and do not present with restrictive filling pattern or heavily infiltrated hearts.^4^ Patients with frequent palpitations, syncopal episodes and AF might be referred to cardiologists. In these setting, young age, non-severely dilated atria and need for multiple cardioversions because of frequent arrhythmia recurrence may suggest the presence of underlying ATTR-CA. ECG recordings or Holter monitoring can detect bradyarrhythmias such as paroxysmal or persistent atrioventricular (AV) blocks and intraventricular conduction delays (i.e. trifascicular block) leading to pacemaker (PM) implantation in 9% of patients within 3 years from the diagnosis.^29^ ATTR-CA has an established association with aortic valve stenosis, although whether amyloidosis is the cause or consequence of valve disease is not fully elucidated.^16^ The progressive reduction in stroke volume observed in ATTR-CA leads to higher frequency of low-flow low-gradient aortic valve stenosis compared to the general population.^16^

Symptoms are mostly nonspecific, including fatigue, dyspnoea, weight loss, peripheral oedema, bleeding tendency (in ATTRv amyloidosis), and neurological manifestations.^24^ Useful clues to diagnosis include a diagnosis of HCM after the sixth decade, history of unexplained neuropathic pain (including prior CTS), need for down-titration or discontinuation of antihypertensive therapy due to poor tolerability, orthostatic hypotension, vitreous opacities and bowel dysfunction (*Table 2*).^24^ Periorbital bruising and macroglossia can be present, but they are far more common in AL amyloidosis.^24^

**Table 2:** Clinical, laboratory, and imaging red flags for ATTR amyloidosis

Clinical	Hypertrophic cardiomyopathy diagnosed after the sixth decade of life Hypertensive cardiomyopathy diagnosed in elderly patients with normal BP and no valvular disease. Need for down-titration or discontinuation of antihypertensive therapy due to poor tolerability. Intolerance of ẞ-blockade in newly diagnosed heart failure. Bowel dysfunction (constipation, diarrhoea). Heart failure with preserved ejection fraction. Aortic valve stenosis in the elderly. Periorbital purpura and macroglossia. Bilateral CTS, atraumatic rupture of biceps tendon and LS stenosis. Unexplained neuropathic pain, mostly in non-diabetic patients. Orthostatic hypotension and erectile dysfunction due to autonomic neuropathy. Vitreous deposits.
Laboratory	Persistent mild increase in troponin levels on repeated tests. Increased NT-proBNP values (> 5000 pg/mL) in non-advanced heart failure Low serum transthyretin values.
ECG	Low QRS voltages (peripheral and/or precordial leads). Discrepancies between the degree of wall thickening and QRS voltages AV delay and/or blocks. Pseudonecrosis.
Echocardiography	Diffuse left ventricular wall thickening. Coexisting right ventricle hypertrophy. Restrictive diastolic filling pattern. Paradoxical low-flow low-gradient aortic valve stenosis. Pericardial effusion. Increased thickness of the interatrial septum and atrio-ventricular valves. Granular sparkling appearance of the myocardium. Reduced left ventricular longitudinal strain with ‘apical sparing’ pattern.
CMR	Diffuse late gadolinium enhancement with subendocardial or transmural distribution Significant elevation in native T1 values Increased extracellular volume fraction (>0.30).
Bone scintigraphy	Cardiac uptake of bone tracers.
PET	Cardiac uptake of tracers.

ATTR, transthyretin amyloidosis; BP, blood pressure; CMR, cardiac magnetic resonance; CTS, carpal tunnel syndrome; LS, lumbar spine; PET, positron emission tomography.

ATTR amyloidosis, especially ATTRwt amyloidosis, is associated with specific osteo-articular disorders, that strongly raise the suspicion of disease in patients with unexplained LV wall thickening. CTS, resulting from amyloid deposits in the soft tissues with nerve entrapment syndrome, is a typical finding in patients with ATTR amyloidosis that often precedes the diagnosis of ATTR-CA by an average of 5 to 10 years.^4,18^ Although CTS has been reported in up to 60% of patients with ATTR amyloidosis,^18^ mostly in single-centre studies with referral bias, a recent population-based Italian study measured an adjusted prevalence of CTS of 14% in ATTRv amyloidosis and 25% in ATTRwt amyloidosis compared to the general population.^30^ In this study, the incidence of CTS in AL amyloidosis was comparable to that observed in the general population.^30^ Although CTS is recognized as an early marker of future amyloidosis, a significant percentage (≈10%) of patients already harbours amyloid deposits in their tenosynovial tissue at the time of carpal tunnel surgery and, among them, 20% have established, undiagnosed ATTR-CA.^24^

Patient with ATTR amyloidosis may present with lumbar spinal stenosis, which results from the deposition of amyloid in the ligamentum flavum causing compression and narrowing of the spinal canal. Histological evidence of amyloid has been reported in 45 to 96% of older patients undergoing spinal stenosis surgery.^24^ Atraumatic rupture of the distal biceps tendon, producing the peculiar ‘Popeye sign’, and rotator cuff injury requiring surgical treatment can be found in 33 and 38% of patients with ATTR amyloidosis, respectively.^24^ Similarly, arthroplasty due to hip and knee disease is more frequent (3 to 5 times) in patient with ATTR amyloidosis than age- and sex-matched controls.^24^

In patients with unexplained LV wall thickening, the above mentioned extracardiac findings have to be carefully investigated as they strongly orient towards the suspicion of ATTR amyloidosis, which have to be confirmed with further testing (*Table 2*).

## 7. Diagnosis

### 7.1 Noninvasive testing for ATTR-CA

**Figure 2:**
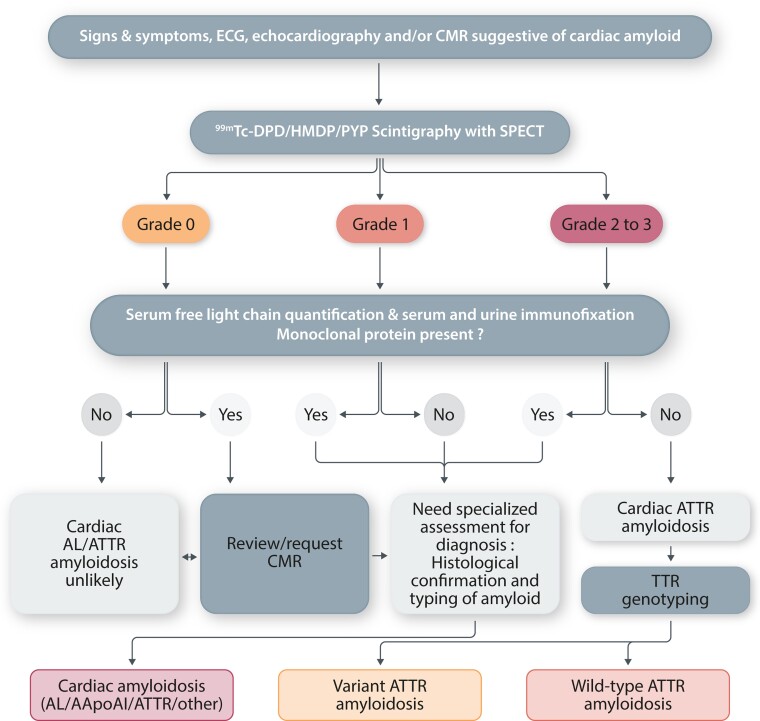
Diagnostic algorithm for patients with suspected cardiac amyloidosis. 99mTc-DPD, 99mTc-3,3-diphosphono-1,2-propanodicarboxylic acid; 99mTc- HMDP, 99mTc-hydroxymethylene diphosphonate; 99mTc-PYP, 99mTc-pyrophosphate; AApoA1, apolipoprotein A-I; AL, light chain; CMR, cardiac magnetic resonance; SPECT, single-photon emission tomography imaging; TTR, transthyretin. Readapted with permission from Gillmore et al^8^.

Historically, the gold standard for the diagnosis and subtyping of ATTR-CA has been EMB.^24^ However, wide use of this technique has been limited by procedural risks, restricted access to experienced centres and availability of pathologists with specific expertise in the cardiovascular field to accurately interpret the histological findings.^31^ Furthermore, the heterogeneous and patchy deposition of amyloid in the heart makes EMB an unreliable method to track disease evolution over time.^31^ In recent years, a landmark study by Gillmore *et al*.^8^ paved the way for the clinical application of cardiac scintigraphy with bone tracer for the non-invasive diagnosis of ATTR-CA,^8^ without the need for histological documentation of cardiac amyloid (*Figure 2*).

In this evolving scenario, a contemporary approach to diagnosis of ATTR-CA includes (i) collection of family history of amyloidosis, ancestries and geographical area of origin:; (ii) clinical examination with a cardiomyopathy-oriented interpretation of findings; (iii) serum biomarker testing, including B-type natriuretic peptide (BNP or N-terminal pro-BNP) and cardiac troponin (T or I); (iv) electrocardiogram (ECG); (v) echocardiography with strain imaging; (vi) CMR; (vii) cardiac scintigraphy with bone tracers; (viii) biochemical testing for a monoclonal protein in serum or urine; and (ix) genetic testing for TTR variants.

#### 7.1.1 Cardiac biomarkers

Circulating biomarkers reflect the pathophysiology of amyloidosis and are related to (i) the amyloidogenic cascade and (ii) the presence and degree of organ involvement. Serum natriuretic peptides and troponin are commonly elevated as amyloid deposition in the heart damages cardiomyocytes.^32^ Therefore, careful evaluation of these biomarkers is important for screening patients for ATTR-CA, risk stratification, response to therapy and treatment tailoring (*Figure 1*). Persistent elevation in serum troponin values in patients with increased wall thickness on echocardiography raises the suspicion of disease, especially in absence of hypertension or aortic valve stenosis.^4^ Furthermore, disproportionate circulating levels of natriuretic peptides to the clinical severity of HF signs and symptoms is suggestive of CA and should prompt further testing to elucidate the underlying cause.^4^

TTR and RBP are biomarkers of the amyloidogenic cascade. TTR has a half-life of ≈2 days and a normal serum level of 0.20–0.45 mg/dL. A number of factors can affect circulating TTR levels including, but not limited to, age, sex, inflammatory disorders, liver disease, chronic kidney disease, hyperthyroidism and malnutrition.^32^ Laboratory methods used for quantification of serum TTR levels do not differentiate wild-type from variant TTR and genetic testing is required to identify TTR mutations. Variant TTR is commonly a less stable circulating tetramer than wild-type TTR and tends to infiltrate tissue with subsequent reduction in TTR serum levels.^24^ This phenomenon represents the rationale for the potential clinical use of serum TTR as a screening biomarker for ATTRv amyloidosis and as a tool to assess response to treatment in both ATTRwt and ATTRv amyloidosis. Serum TTR levels are increased by TTR stabilizers (i.e. tafamidis, acoramidis) and decreased by TTR ‘gene silencers’ such as siRNA and ASO (i.e. patisiran, inotersen).^4^ RBP is the most powerful natural stabilizer of circulating TTR.^33^ The ability of this ligand to bind to TTR can be reduced in presence of *TTR* mutations leading to increased renal clearance and low serum RBP levels, further promoting TTR destabilization.^33^ This phenomenon was observed in cohorts of patients with V122I related ATTR-CA, in which a multi-parametric clinical prediction model integrating serum RBP levels was demonstrated useful for diagnosing V122I ATTRv-CA.^33^

Evaluation of biomarkers is of particular value in patients at higher risk for developing amyloidosis due to the presence of monoclonal gammopathy of unknown significance (MGUS) or *TTR* gene mutations. When interpreting natriuretic peptide and troponin levels, clinicians should consider the presence of impaired renal function and AF that affect circulating levels of these biomarkers.

#### 7.1.2 Electrocardiography

Even in the contemporary era of advanced cardiovascular imaging and deep non-invasive phenotypic characterization, the electrocardiogram (ECG) retains an important role in the assessment of patients with suspected ATTR-CA. Traditionally, low voltage QRS complexes, defined as a QRS amplitude <5 mm in the limb leads or <10 mm in the precordial leads, have been considered the electrocardiographic hallmark of CA.^24^ However, their prevalence varies considerably, ranging from 27% in ATTR-CA to 84% in AL-CA. The established linear relationship between LV mass and QRS voltage on the surface ECG does not hold true in CA as the increase in wall thickness is caused by amyloid deposition rather than true cardiomyocyte hypertrophy. Only ∼50% of patients with AL-CA and about 25–40% of those with ATTR-CA meet true low voltage QRS criteria.^24^ Therefore, the absence of low voltage QRS complexes does not rule out a diagnosis of CA and should not discourage clinicians from considering the diagnosis. Low voltage QRS complexes reflect the loss of vital myocardium due to diffuse amyloid deposition in the heart. In clinical practice, they can precede the increase in LV wall thickness or coexist with severe concentric biventricular hypertrophy, thus resulting from the interplay of multiple factors, not entirely elucidated. For example, a greater relative increase in cardiomyocyte hypertrophy has been found in ATTR compared to AL-CA, providing an explanation for the presence of normal QRS complexes and voltage criteria for LV hypertrophy in 44 and 25% of patients with ATTR and AL-CA, respectively.^34,35^ In addition, the presence of myocardial oedema, predominantly in patients with AL-CA, is a previously underappreciated factor that might explain the higher frequency of low voltage QRS in AL compared to ATTR-CA, despite a typically greater amyloid burden in the latter.^24^

Beside low voltage QRS complexes, other electrocardiographic abnormalities characterize the majority of patients with ATTR-CA. A pseudoinfarct pattern with Q waves and poor R wave progression in the precordial or limb leads can be found in 50% of cases.^24^ Bradyarrhythmias and conduction system diseases are common and might be caused by progressive amyloid infiltration and autonomic dysfunction, especially in ATTRv-CA and AL-CA. AF is the most frequent arrhythmia in ATTR-CA, affecting 45–70% of patients, due to atrial dysfunction resulting from extensive amyloid infiltration within the myocardial interstitium.^36^ Atrial myocytes bundles are progressively isolated with significant intra-atrial conduction delay leading to longer cycle lengths and a more organized AF on surface electrocardiogram.^24^ Sinus node dysfunction can occur in 7% of patients with CA.^24^ First-degree AV block can be found in 35–50% of patients with ATTR-CA.^24^ Second-degree and third-degree AV blocks are common and lead to PM implantation during the course of the disease.^24^

The ECG is a widely accessible and inexpensive test that provides valuable information to raise the suspicion of disease, orient the following phases of the diagnostic work-up and guide appropriate decision-making.

#### 7.1.3 Echocardiography

Echocardiography is the first line imaging modality that most frequently raises the suspicion of CA, detecting typical signs (‘red flags’) of an underlying infiltrative cardiomyopathy.^2^ Although amyloid deposition can occur at any site in the heart, the process is most marked in the ventricular walls. Therefore, the hallmark of cardiac amyloid deposition is concentric LV or biventricular thickening (>1.2 cm) in the absence of aortic valve disease or significant systemic hypertension, although CA, particularly AL-CA, may present with normal LV mass. In this model of cardiomyopathy, the increased wall thickness is a ‘pseudo-hypertrophy’ related to a progressive infiltration of amyloid in the myocardium rather than a true cardiomyocyte hypertrophy.^4,24^ This is the mechanism underlying the characteristic discrepancy between low voltage QRS complexes and increased wall thickness and cardiac mass observed in CA.

**Figure 3:**
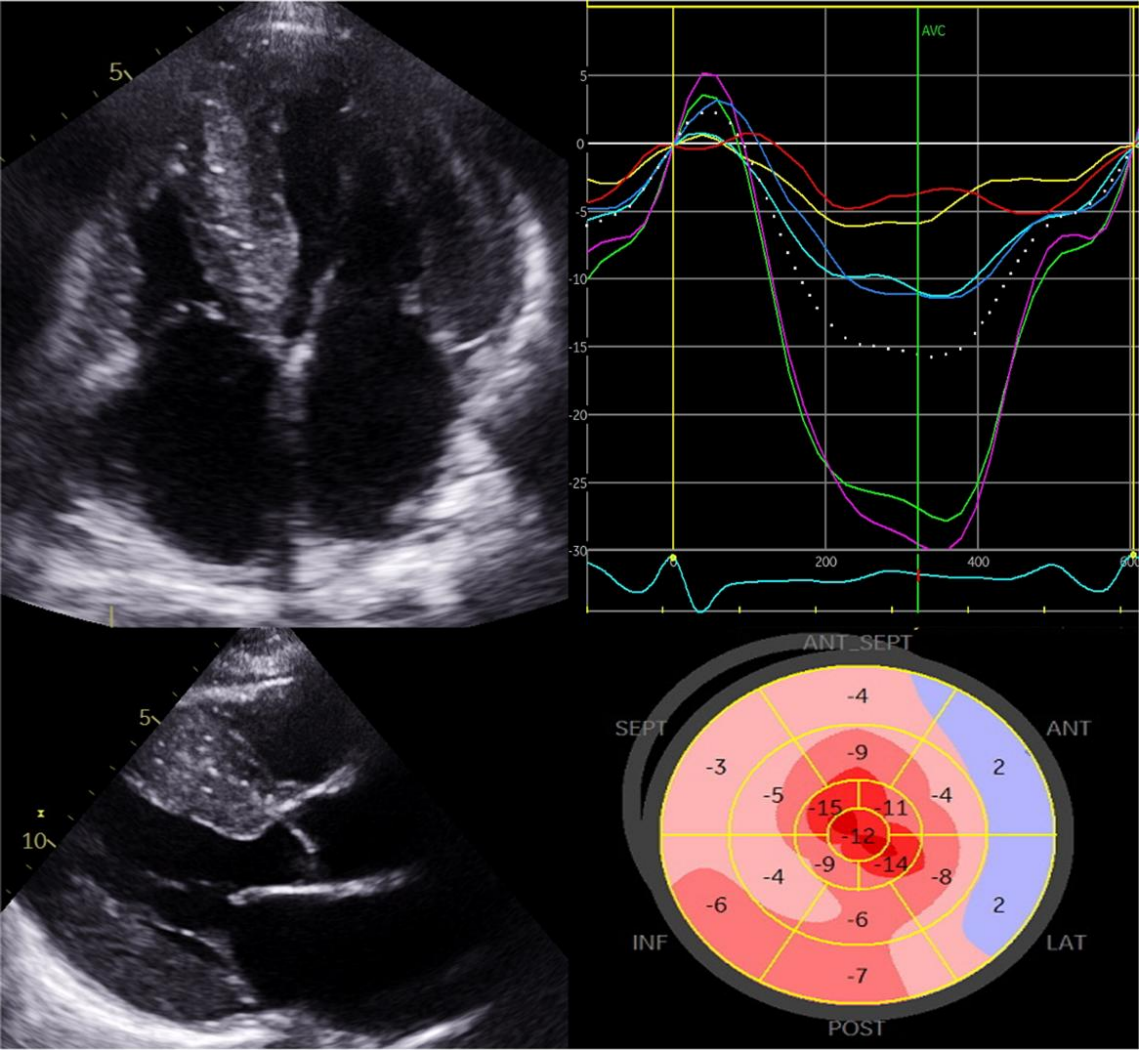
Echocardiographic findings in a patient with advanced transthyretin cardiomyopathy. Parasternal long axis and four-chamber view demonstrating increased biventricular thickening with a ‘speckled’ myocardium (top left panels) with 2D-strain using speckle tracking echocardiography in the same patient (top right panel). Peak systolic strain for individual myocardial segments in the four-chamber view panel and the strain curve samples in each of the corresponding coloured myocardial segments panel can then generate a longitudinal strain map (bottom). The characteristic basal to apical gradient of impaired longitudinal function is observed here.

Classical echocardiographic findings of CA include thickened ventricles with a ‘granular speckling’ appearance of the myocardium, small LV chamber volume, atrial dilatation, restrictive diastolic filling due to noncompliant ventricles, interatrial septal thickening, valve thickening, prominent RV wall thickening and pericardial effusion (*Figure 3*).^37^ The majority of these findings have a low accuracy for diagnosing CA, mostly due to low sensitivity. Myocardial contraction fraction (MCF), measured as ratio of stroke volume to myocardial mass, is the conventional echocardiographic parameter with the best diagnostic accuracy (AUC of 0.80).^37^

Over time, progressive amyloid deposition determines an increase in myocardial mass and a progressively smaller ventricular cavity size with low stroke volume.^37^ These mechanisms establish a condition of fixed end-diastolic volume where cardiac output becomes critically dependent on heart rate. The raise in ventricular filling pressures, reflected by increased E/e’ ratio, causes a progressive increase in atrial dimensions, although severe dilatation is usually prevented by thickened, stiff atrial walls infiltrated by amyloid.^37,38^ Furthermore, atrial function is significantly impaired in all components (reservoir, conduit and contraction) during the entire cardiac cycle, resulting in a condition of ‘electromechanical dissociation’ in ≈20% of patients in sinus rhythm.^36^

CA is regarded as a cause of HFpEF as ejection fraction (EF) tends to be preserved up until higher burdens of cardiac infiltration. However, EF does not reliably reflect systolic function in CA.^37^ Longitudinal function is typically affected before radial contraction as reflected by the impairment in mitral and tricuspid annular plane systolic excursion (MAPSE and TAPSE), despite a normal EF.^37^

Longitudinal strain (LS) measured by speckle tracking echocardiography provides more accurate estimation of longitudinal function and can aid differentiating CA from other causes of wall thickening such as hypertension and HCM. Regional LV strain analysis demonstrated a LS gradient with more severe impairment in the basal segments and a relative preservation at the apex. This feature gives rise to a characteristic ‘bulls-eye’ pattern on strain plotting that is sensitive (92%) and specific (82%) in discriminating CA from others causes of LV hypertrophy and carries a poorer prognosis.^2,38^ The mechanism underlying the apical sparing of LS remains unclear, although relatively reduced amyloid deposition at this level, peculiar myocardial fibre orientation and different conditions of parietal stress and blood flow have been proposed.^2,37^

The echocardiography laboratory represents the crossroad where screening for CA can be systematically performed with a ‘red-flag’ approach.^23^ Of note, the single threshold of a wall thickness >1.2 cm is not sex-specific and, if used in isolation, confers a high degree of specificity, but low sensitivity for the diagnosis of CA. For this reason, recent international statements and position papers on amyloid disease recommended ascertainment of CA in subjects with increased wall thickness in combination with other clinical and instrumental suggestive red flags.^39^ A scoring system using specific echocardiographic parameters with highly sensitive and specific cut offs was proven useful for confirmation or exclusion of CA.^40^ Second-level imaging modalities were recommended in patients with intermediate probability of disease. Although not yet externally validated, a score ≥8 points had a high diagnostic accuracy for AL and ATTR-CA.^40^ In addition, the combination of echocardiographic and clinical information has been recently demonstrated to enhance the opportunity to diagnose CA in patients ≥55 years with non-dilated LV, a wall thickness >1.2 cm hearts and preserved LVEF. In these subjects, the presence of at least 1 red flag of CA in combination with easily-obtainable clinical information, such as age, arterial hypertension and bilateral CTS, resulted an accurate approach in screening patients to raise the suspicion of CA and in orienting specific diagnostic work-up in the general population.^23^

Whilst echocardiography is highly informative to raise the suspicion of disease, it is not possible to differentiate AL and ATTR-CA by echocardiography alone and additional tests are needed to reach a final diagnosis.

#### 7.1.4 CMR imaging

CMR has revolutionized the non-invasive work up of cardiomyopathies, particularly in CA. This imaging technique provides detailed tissue characterization and comprehensive information on cardiac structure and function, differentiating CA from other cardiac diseases with hypertrophic phenotype. The use of CMR to assess the heart of patients with CA has increased significantly our understanding of this condition.^34^ Concentric and symmetric hypertrophy was considered a typical finding in CA as opposed to asymmetric wall thickening in HCM. However, CMR studies revealed that the most common phenotype of patients with ATTR-CA is asymmetrical LV hypertrophy (≈80% of cases), with similar findings in ATTRwt-CA and ATTRv-CA, while symmetrical and concentric LV hypertrophy can be found in 18% of cases.^41^

**Figure 4:**
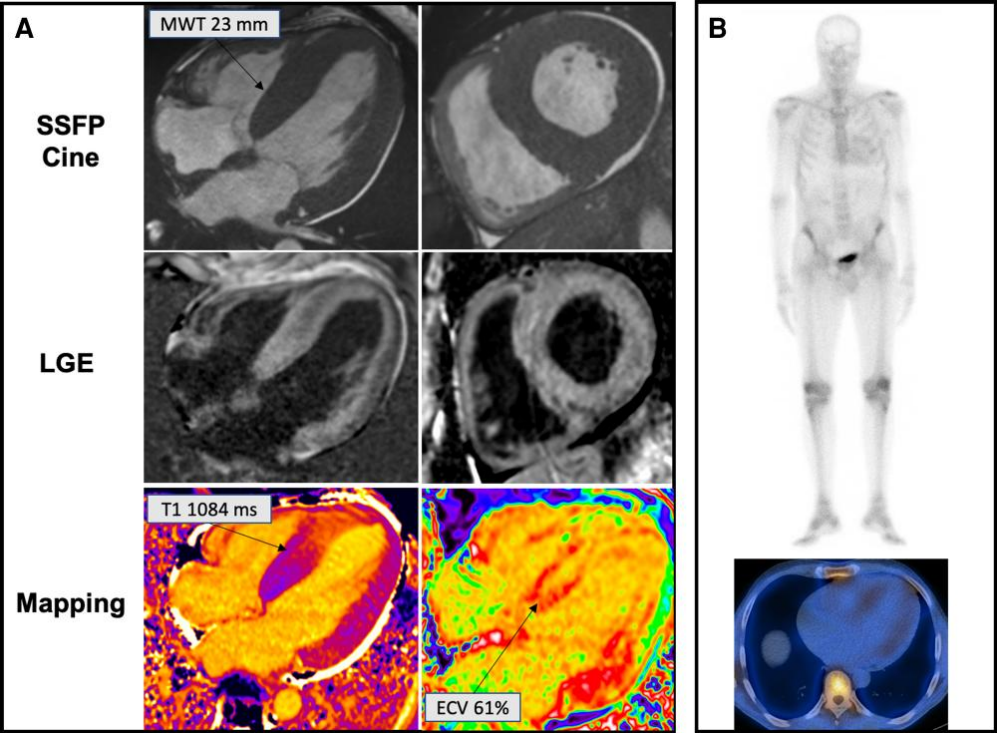
A 63-year-old gentleman with lumbar canal stenosis and a 3-month history of exertional breathlessness diagnosed with transthyretin cardiac amyloidosis associated Ser97Tyr variant. Legend: Panel A. Top raw: increased wall thickness on steady-state free precession cine imaging. Middle raw: transmural late gadolinium enhancement with marked involvement of the right ventricle. Bottom raw: High native T1 value and extracellular volume fraction. Panel B: Perugini grade 1 abnormal cardiac uptake of bone tracer on planar (top) and single-photon emission tomography (bottom) imaging.

The key advantage of CMR in CA is its unique ability to give information about the tissue composition by ‘myocardial tissue characterization’^42^ (*Figure 4*). The administration of extracellular gadolinium-based contrast agents that cannot cross the intact myocyte cell membrane and accumulate in the gaps between cells allows to visualize the extracellular matrix expansion resulting from amyloid fibril deposition with the late gadolinium enhancement (LGE) technique and the contrast-enhanced T1-weighted imaging for the calculation of extracellular volume (ECV).^2^ Under normal conditions, cardiomyocytes are densely packed and represent the greater part (∼85%) of the myocardial volume and the normal ECV is usually in the range of 22 to 28%.^42^ CA is a pure primary interstitial process that results in a progressive expansion of the extracellular space with increased gadolinium concentrations in the myocardium and, therefore, hyper-enhancement. For this reason, CA has a highly characteristic appearance on LGE imaging, with initially diffuse sub-endocardial LGE that becomes transmural in the later stages of the disease, coupled with abnormal gadolinium kinetics with the myocardium and blood nulling at the same time.^41^ Interpretation of LGE imaging can be challenging in light of the diffuse myocardial amyloid deposition, which makes it more difficult to determine the optimal null point for the myocardium.^42^ Traditional LGE imaging is a thresholding or comparison technique that requires adequate nulling of the normal myocardium by an expert operator. Phase-sensitive inversion recovery (PSIR) approach overcomes this because the PSIR reconstruction cannot erroneously null the tissue with the shortest T1, making the technique less operator dependent.^2^

Patients with CA can exhibit 3 different LGE patterns (no LGE, subendocardial LGE and transmural LGE) that have good correlation with the degree of myocardial infiltration (86% sensitivity, 92% specificity).^41^ The degree of LGE transmurality (i.e. from subendocardial through to transmural) reflects the underlying infiltrative process and is an important marker of all-cause mortality.^42^ However, evaluation of LGE requires administration of gadolinium-based contrast agents (not feasible in patients with renal impairment) and LGE imaging is a non-quantitative technique, limiting its role in quantifying the amyloid burden.^2^

T1 mapping offers a quantitative pixel-based measure of the myocardial T1 relaxation time—either pre-contrast (native) or post-contrast—and can overcome both these limitations. Native myocardial T1—the intrinsic signal from the myocardium—progressively increases in CA and tracks markers of systolic and diastolic function, as well as cardiac amyloid infiltration, from early stages to massive diffuse transmural involvement.^43^ Native myocardial T1 elevation can be observed even before LGE development and is associated, in single centre studies, with a high diagnostic accuracy for CA, in settings with high pre-test probability.^43,44^ Recently, a diagnostic algorithm based on native myocardial T1 values has been developed to confirm CA in patients with clinical suspicion of disease,^44^ paving the way for the use of this parameter in patients with renal imparment in whom the benefits of gadolinium contrast administration must be carefully balanced against the risks. Native T1 and ECV have both been validated as markers of amyloid infiltration in the heart and were demonstrated to correlate with the degree of cardiac infiltration measured by 99mTc-3,3- diphosphono-1,2-propanodicarboxylic acid (99mTc-DPD) scintigraphy.^35^ Interestingly, changes in these parameters have been reported in different types of amyloid: higher native myocardial T1 in AL-CA and greater ECV in ATTR-CA. Native T1 is a composite myocardial signal from both interstitium and myocytes and does not differentiate fully the underlying processes (fibrosis, oedema, amyloid, myocyte response). Processes not related to amyloid infiltration such as myocardial oedema (frequently found in AL-CA) and cardiomyocyte hypertrophy (predominantly seen in ATTR-CA) can affect this parameter.

The ratio of pre and post contrast T1 maps can be used in combination with the patient’s haematocrit to isolate the signal from the extracellular space and to obtain a surrogate quantitative measure of cardiac amyloid burden: the ECV.^2^ Therefore, this parameter more accurately reflects true amyloid infiltration in the heart.^35^ Massive elevation in ECV fraction (values greater than ≈0.4 in the remote myocardium) are characteristic of CA.^2^ Elevation of ECV is an early disease marker, before the development of LGE, and correlates with other markers of disease severity, including cardiac biomarkers and changes in cardiac morphology on echocardiography.^2^ This parameter can track disease severity across the spectrum of amyloid infiltration and correlates independently with prognosis in both types of amyloidosis.^35,45^ In addition, ECV can quantify the burden of splenic and hepatic amyloid in systemic amyloidosis without the need for any additional imaging sequences.^46^ Uniquely, this parameter has been shown to be able to track response to treatment at cardiac and extracardiac level, in both AL and ATTR amyloidosis.^34,47,48^

Another useful parameter for full myocardial characterization in CA is T2 mapping that is a sensitive method to detect myocardial oedema in ischaemic and non-ischaemic cardiomyopathies. The highest T2 values have been reported in cohorts of untreated patients with AL-CA compared to those with treated AL and ATTR-CA. Of note, increased T2 values (> 55 ms) have been associated with mortality in AL-CA.^49^ Therefore, this parameter has the potential to track disease progression and response to therapy. The presence of myocardial oedema provided additional information of the multiple pathophysiological mechanisms of myocardial damage involved in CA.^49^

In summary, CMR has become an important technique in the evaluation of CA at different clinical stages: diagnosis, prognostic stratification and response to therapy (*Table 3*). Although it provides comprehensive information about myocardial tissue composition, none of the above-mentioned CMR parameters can be used to reliably differentiate AL from ATTR-CA.

**Table 3:** Comparisons of benefits and limitations between transthoracic echocardiography, cardiac magnetic resonance, and bone scintigraphy

	TTE	Bone scintigraphy	CMR
Cost^a^	$	$$$$	$$$$$$$$
Accessibility	Widely available	Centres with gamma camera access.	Tertiary cardiac centres.
Cannulation	Not routinely required, required for contrast echocardiography.	Required for radioactive tracers.	Required for gadolinium-based contrast agents.
Biological damage	No	Yes	No
Duration	20 min	Scan duration of 30 min performed 2–4 h post tracer injection.	45–60 min
Advantages	Safe in pregnancy	Not operator-dependent Unaffected by body habitus. Discriminate between ATTR and AL amyloidosis.^b^ Detect cardiac involvement in early disease stages.	Can distinguish ATTR-CA from other heart muscle diseases. Full tissue characterization. Potential use to monitor disease evolution and treatment response.
Limitations	Tissue characterization not available Image quality affected by operator and body habitus	Not safe in pregnancy or breastfeeding. Time-consuming. Exposure to ionizing radiation.	Time-consuming. Injection of contrast agents not feasible in renal impairment. Suboptimal image quality in arrhythmias and poor breathing).

AL; light chain; ATTR-CA; transthyretin cardiac amyloidosis; CMR; cardiac magnetic resonance; TTE; transthoracic echocardiography.

Costs estimates are taken from NHS tariffs (2020/21) and may differ in different countries.

In combination with search for monoclonal proteins in serum and urine.

#### 7.1.5 Nuclear scintigraphy

##### 7.1.5.1 Bone-Avid compounds

The first report using myocardial uptake of bone tracers on planar scintigraphy in patients with proven CA dates back to early 1980s.^2^ The mechanism responsible for myocardial uptake remains a grey area, although it might be related to cardiac microcalcifications^50^—more abundant in ATTR than AL-CA—or to amyloid fibril composition (type A and B).

**Figure 5:**
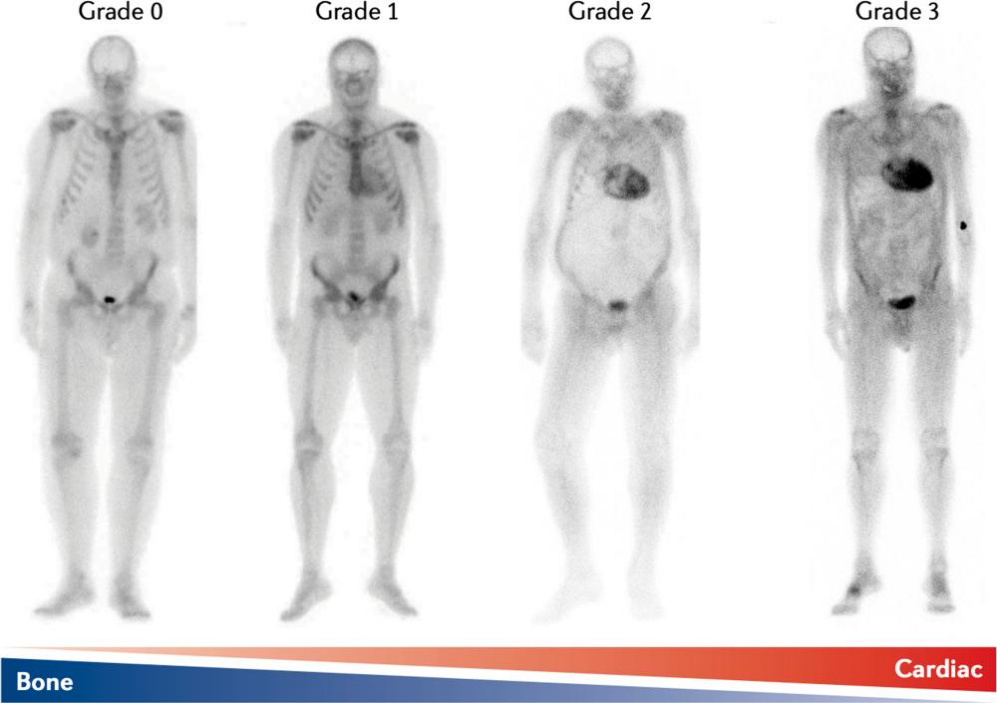
Spectrum of cardiac uptake of 99mTc-3,3-diphosphono-1,2-propanodicarboxylic acid (99mTc-DPD) on planar scintigraphy. Legend: Grade 0: no cardiac uptake; Grade 1: mild cardiac uptake, less than bone uptake; Grade 2: moderate cardiac uptake accompanied by attenuated bone uptake; Grade 3, strong cardiac uptake with mild/absent bone uptake. Legend: AApoAI, ApoAI amyloidosis; AL, light chain; ATTR, transthyretin amyloidosis; CMR; cardiac magnetic resonance; ECG, electrocardiography; SPECT; single-photon emission computed tomography; TTR, transthyretin.

Technetium-labelled radioactive tracers currently approved for non-invasive confirmation of ATTR-CA with similar diagnostic performance are 99mTc-pyrophosphate (99mTc-PYP), 99mTc-hydroxymethylene diphosphonate (99mTc- HMDP), and 99mTc-DPD.^51^ The presence and degree of cardiac retention of bone tracer can be assessed by qualitative and quantitative methods. The most common qualitative method is the Perugini score (*Figure 5*) that grades myocardial uptake in relation to bone uptake on planar images: grade 0 = no cardiac uptake; grade 1: mild cardiac uptake, less than bone uptake; grade 2: moderate cardiac uptake accompanied by attenuated bone uptake; grade 3, strong cardiac uptake with mild/absent bone uptake.^52^ Quantitative methods include the heart to contralateral (H/CL) ratio on 99mTc-PYP planar images and the heart to whole body (H/WB) ratio on 99mTc-DPD and 99mTc-HMDP planar images.^52^

Cardiac scintigraphy with bone tracers is the cornerstone of an imaging-based diagnostic pathway for accurate non-invasive confirmation of ATTR-CA that has been recommended by national and international scientific societies (*Figure 2*). This imaging modality may reliably differentiate CA from other cardiac diseases presenting with wall thickening such as HCM. However, the presence of myocardial uptake in isolation does not confirm the diagnosis of ATTR-CA since 40% of patients with AL-CA have cardiac uptake (including Perugini grade 2 or 3 positivity in ≈10%) and cardiac apolipoprotein A-I amyloidosis is associated with low grade (Perugini grade 1) myocardial uptake.^8^ In the presence of suggestive clinical, echocardiography or CMR findings, the positive predictive value of Perugini grade 2 or 3 myocardial uptake approaches 100% for the diagnosis of ATTR-CA as long as there is no evidence of any serum or urine monoclonal protein by all of three biochemical assays: serum immunofixation, urine immunofixation, and serum free light chain assay.^8^ This algorithm is based on the results of a landmark study by Gillmore *et al*.^8^ and reliably establishes the diagnosis of ATTR-CA without the need for tissue biospy in ≈70% of cases. Histological confirmation and typing of amyloid remain essential in patients who do not meet the non-invasive diagnostic criteria for ATTR-CA outlined above.^31^

Of note, the population enrolled in that study did not encompass the full spectrum of TTR pathogenic mutations. Low sensitivity of cardiac scintigraphy with bone tracers has been reported in patients with biopsy-proven ATTR-CA related to rare TTR variants such as Ser97Tyr, Tyr114Cys and Phe64Leu (*Figure 4*).^41,53^ Caution should also be applied when using cardiac scintigraphy with bone tracers since false negative scans may occur in association with other rare, as yet unidentified, vATTR-CA syndromes. Therefore, a negative scan in patients with strong clinical suspicion of ATTR-CA does not exclude the diagnosis and should be integrated by advanced imaging modalities, such as CMR, and, in selected cases, by EMB.^31^ In addition, expert recommendations^52^ on cardiac scintigraphy with bone tracer recommend the systematic use of single photon emission computed tomography (SPECT) imaging to enable better understanding of bone tracer localization. Given that these tracers are also blood pool agents, SPECT imaging can determine if uptake is in the myocardium (consistent with CA), in the ventricular cavity (blood pool) or outside the heart (sternum or rib).^52^

While myocardial uptake (measured as Perugini visual score, H/CL or H/WB ratio) has gained diagnostic value over time, its prognostic role remains controversial.^2^ Cardiac retention of bone tracer has been associated with major adverse cardiac events, acute HF and increased mortality (i.e. poor survival in presence of an H/CL of ≥1.6).^2^ More recently, a potential association of biventricular uptake with increased all-cause mortality, cardiac death and hospitalization for HF has been reported in patients with ATTR-CA undergoing scintigraphy with planar and SPECT imaging.^52^ Although intriguing, these results have to be confirmed in future dedicated studies. Data on monitoring disease activity through serial scans is limited given differences in bone tracer kinetic and distribution in the body and the peculiar phenomenon of reciprocal changes in the heart, bone, skeletal muscle and soft tissue because of competitive uptake.^54^

Finally, solid evidence in the clinical application and significance of nuclear medicine in amyloidosis is limited by the adoption of bone tracers with different characteristics—PYP in the United States, HMDP in France, DPD in the United Kingdom and Italy—and the heterogeneity in grading systems and imaging protocols of acquisition adopted in Europe and in the United States.^2^

##### 7.1.5.2 Positron emission tomography (PET) agents

PET imaging is an emerging imaging modality holding great promise for diagnosing CA and differentiating AL from ATTR-CA. The cardiac uptake of tracers is probably related to the binding to the β-pleated structure of amyloid fibrils, allowing identification of amyloid deposits with different precursor protein.^2^

Acquisition of late ^18^F-florbetaben PET/CT images using a 30-min dynamic protocol has been associated with a significantly greater cardiac uptake in AL than in ATTR-CA,^55^ supporting the possibility of using this imaging modality for differentiating between these types of CA.

The clinical application of PET imaging is limited by high costs, need for an onsite cyclotron and very limited evidence in CA. Although intriguing, data on PET and CA need to be confirmed in future dedicated large prospective studies.

##### 7.1.5.3 Amyloid-directed scans

Serum amyloid P component (SAP) is a minor constituent of all amyloid deposits. The SAP coating of amyloid deposits is used in ^123^I-labelled SAP (^123^I-SAP) scintigraphy to determine where and how much amyloid has been deposited in certain organs. This imaging technique provides non-invasive quantification of amyloid burden in the liver, spleen and kidneys of patients with amyloidosis. However, hollow or moving organs such as the gastrointestinal tract and the heart cannot be assessed reliably by this technique due to low signal.^2^

In clinical practice, ^123^I-SAP scintigraphy is a useful tool, in diagnosis of patients with AL, serum amyloid A (AA) and hereditary amyloidoses, to provide a whole body overview of visceral amyloid deposition (‘amyloid load’), to quantify the extra-cardiac amyloid burden, to detect organ involvement and identify sites for biopsy, and to monitor changes in amyloid burden and response to treatment over months and years.^2^

## 8. Staging and clinical course

The clinical course of ATTR-CA depends on fibril type (ATTRwt vs. ATTRv amyloidosis), specific mutation, age of onset, severity of cardiac involvement and, potentially, fragmented vs. full-length fibrils. The natural history of disease includes progressive HF, complicated by arrhythmias and conduction system disease. The median overall survival of untreated patients with ATTR-CA is estimated around 3–5 years following diagnosis,^1,56^ with significant differences in ATTRv amyloidosis according to the specific TTR variant: overall 4-year survival of 16% in V122I, 40% in T60A and 79% in V30M. Whilst ATTRwt amyloidosis is a more homogeneous model of disease affecting predominantly elderly patients presenting with HFpEF, ATTRv amyloidosis has a polymorphic clinical presentation as some specific TTR mutation may predispose to a more severe cardiac involvement (i.e. V122I).^5^ Prognostic stratification in ATTR-CA remains challenging, as the diagnosis is made at different stages of the disease and the risk is dynamic over time due to progressive amyloid deposition in the heart. Although major advances have been made in disease recognition, a significant proportion of patients are still diagnosed many years after disease onset and has commonly been managed as having HCM or other phenocopies.^4^ In recent years, an increasing number of parameters have proven useful for risk prediction in ATTR-CA including advanced NYHA functional class, cardiac biomarkers, low voltage QRS complexes on surface ECG, maximal LV wall thickness, MCF, systolic function, global longitudinal strain on echocardiography, LGE, T1 mapping and ECV on CMR, and, potentially, patterns of cardiac uptake on scintigraphy with bone tracers (*Table 4*).

**Table 4:** Echocardiography and CMR parameters associated with poorer prognosis in ATTR-CA

Parameters	Prognostic thresholds
Parameters—echocardiography	
ȃGLS	≥ −12%
Apical/basal strain ratio	≥2.1
ȃMAPSE	<9 mm
ȃTAPSE	<15 mm
ȃMCF	<0.26
Parameters—CMR (1.5 T)	
ȃNative T1 (shMOLLI)	>1044 ms
ȃLGE	Transmural
ȃECV	>0.45

ATTR-CA, transthyretin cardiac amyloidosis; CMR, cardiac magnetic resonance; ECV, extracellular volume; GLS, global longitudinal strain; LGE, late gadolinium enhancement; MAPSE, mitral annular plane systolic excursion; MCF, myocardial contraction fraction; shMOLLI, shortened modified Look-Locker inversion recovery; TAPSE, tricuspid annular plane systolic excursion.

Following the development of disease-modifying treatments able to improve survival, the life span of patients with ATTR-CA is expected to increase significantly compared to the past.^9^

### 8.1 Baseline

Several risk prediction models based on serum biomarkers have been validated for prognostic stratification in ATTR-CA and include NT-pro-BNP, cardiac troponin, and estimated glomerular filtration rate (*Table 5*). NT-proBNP has an independent prognostic value and changes over time are useful to follow disease progression.^32^ There are 2 classification systems based on NT-proBNP and troponin that, adopting different cut off values, divide patients with ATTR-CA into three stages with increasing mortality. The estimated risk of mortality is classified as high (stage C or I, both biomarkers above the cut-off), intermediate (stage B or II, only one biomarker above the cut-off) or low risk (stage A or III, both biomarkers below the cut-off). Kristen *et al*.^57^ proposed a score combining natriuretic peptides (BNP > 195 ng/L or NT-proBNP > 2584 ng/L) and troponin (TnT > 50 ng/L or TnI > 580 ng/L) in patients with both ATTRwt and ATTRv-CA.^32^ Later, Grogan *et al.*^58^ developed a staging system based on troponin T (50 ng/L) and NT-proBNP (3000 ng/L) in a cohort of patients affected only by ATTRwt-CA.^32^ Recently, Gillmore *et al*.^56^ devised a predictive score—NAC ATTR staging system—based on NT-proBNP (3000 ng/L) and GFR (45 mL/min/1.73 m^2^) for patients with both ATTRwt and ATTRv-CA. Median survival of patients was 69.2, 46.7 and 24.1 months from stage I to III. This score demonstrated a better accuracy for outcome prediction compared to staging systems based on natriuretic peptides and troponin.^59^ Of note, the NAC score accounted also for the effect of TTR variant on survival and reported a poor prognosis in patients with V122I TTR across all stages compared to ATTRwt-CA. Furthermore, a significant advantage of the NAC score is the absence of troponin which has great heterogeneity across centres with regard to the specific type (i.e. type I and T), assay accuracy and methods for quantification (i.e. normal vs. high-sensitivity), limiting the clinical application of the other staging systems. These scores have been validated only in newly diagnosed patients with ATTR-CA before the initiation of specific treatments, but initial data suggest the ability of changes in the NAC stage to predict survival throughout the disease natural history.^60^ The usefulness of these predictive models during follow up is currently an area under investigation.

**Table 5:** Biomarker-based staging and prognosis in ATTR amyloidosis

Model	Type	Biomarker, cut-off	Stages	Estimated median OS (months)
Kristen *et al*.^57^	ATTRwt and ATTRv	NT-proBNP >2584 ng/L (or BNP >195 ng/L) TnT >50 ng/L (or TnI >580 ng/L)	A: 2 >cut-offs	40% alive^a^
			B: 2 <cut-offs	98% alive^a^
			C: 1 >cut-off	80% alive^a^
Grogan *et al*.^58^	ATTRwt	NT-proBNP ≥3000 ng/L TnT ≥50 ng/L	I: both below the cut-offs	66
			II: 1 above the cut-off	40
			III: both above the cut-offs	20
Gillmore *et al.*^56^	ATTRwt and ATTRv	NT-proBNP >3000 ng/L eGFR <45 mL/min/1.73 m^2^	I: both below the cut-offs	69
			II: 1 above the cut-off	47
			III: both above the cut-offs	24

ATTRwt, wild-type transthyretin amyloidosis; ATTRv; variant transthyretin amyloidosis; BNP, B-type natriuretic peptide; eGFR, estimated glomerular filtration rate; NT-proBNP, N-terminal fraction of pro–B-type natriuretic peptide; (hs-) TnT, (high-sensitivity) troponin T; (hs-)TnI, (high-sensitivity) troponin I.

3-year survival estimate.

Finally, TTR is a promising biomarker of the amyloidogenic cascade under evaluation for prognostic stratification. According to initial data on ATTRwt-CA, low serum TTR levels with a cut off value of <18 mg/dL is associated with a poorer survival and decrease in TTR levels is paralleled by worsening cardiac function.^61^ Further research is required to move TTR from bench to bedside.

### 8.2 Disease progression and treatment response

Disease progression in ATTR-CA can be defined according to changes in clinical, biomarkers and imaging parameters. However, monitoring disease evolution and quantifying the impact of specific therapies is challenging as the improvement in clinical features and outcomes following initiation of disease-modifying treatment is not necessarily paralleled by structural changes in the heart. Changes in NT-proBNP (>30% and >300 pg/mL from baseline) and NYHA functional class (2 class improvement from baseline) can provide useful information in ATTR-CA, but they have been validated for assessment of disease evolution only in AL-CA.^32^

Many echocardiography and CMR parameters can provide information on functional and structural abnormalities caused by progressive cardiac amyloid deposition.^35,38^ Recent imaging studies quantifying cardiac amyloid burden by ECV identified three groups of parameters, aside from tissue characterization parameters, that become abnormal sequentially as ATTR-CA progresses.^35,38,41^ Changes in E/e’, cardiac mass, GLS and MAPSE can be observed at earlier stages of amyloid deposition in the heart, while LV SV, MCF, and TAPSE are more likely to be abnormal at intermediate burdens of cardiac infiltration. Finally, deterioration in biventricular ejection fraction and biatrial dilatation occur in late stages of disease. All these parameters are useful to track disease evolution in combination with the degree of LGE transmurality and elevation in ECV.^38^

To date, no robust markers of treatment response have been identified. Diflusinal has been reported to prevent deterioration of GLS on echocardiography compared to placebo.^62^ No substantial improvement in echocardiographic parameters was found in patients with ATTR-CA receiving tafamidis, although stabilization of LV mass, native-T1 and ECV measured by CMR have been reported after one year of treatment in case reports.^62^ In a sub-analysis of the APOLLO trial, improvements in GLS and maximal LV wall thickness on echocardiography were observed after 18 months of treatment in patients taking patisiran,^62^ but changes in these parameters were not found in patients treated with inotersen.^62^ However, myocardial strain analysis is not well standardized and results are affected by changes in cardiac preload and afterload. Therefore, there is a need to identify markers of treatment response at a myocardial level in ATTR-CA.

CMR studies on small cohorts of patients with ATTR-CA reported preservation of wall thickness and overall cardiac mass with inotersen during follow up. The first evidence of amyloid regression in ATTR-CA has been recently reported in patients treated with patisiran. A reduction in ECV measured by CMR over the course of 12 months was found in 38% of patients and was paralleled by scintigraphic, biochemical and functional evidence of clinical benefit. In this study, there was a ≈ 20% reduction in cardiac uptake on DPD radionuclide scintigraphy among patients treated with patisiran, although whether this finding reflects cardiac amyloid regression is not fully elucidated.^48^ Amyloid regression might result from a constant rate of amyloid clearance by biological systems and the reduction in the rate of accumulation of new ATTR amyloid in the heart induced by therapy. Although fascinating, this hypothesis remains unproven. CMR with ECV quantification might be used for monitoring disease response and assessing changes in amyloid burden at an individual level, but further research is needed.^48^ There is a paucity of data on the ability of cardiac scintigraphy with bone tracers and SAP imaging to assess treatment response in ATTR amyloidosis and the use of these imaging modalities for this purpose is controversial.

## 9. Disease-modifying treatments

Advances in biological understanding of the mechanisms involved in TTR amyloid formation over the last years have led to the development of modern therapeutic strategies aimed at reducing the deposition of ATTR in the myocardium through stabilization of the circulating TTR tetramer or reduction in hepatic synthesis of TTR (*Table 6*). Early diagnosis of ATTR-CA and initiation of treatment are crucial to derive the greatest benefit from such therapies and improve outcomes.

**Table 6:** Treatments for ATTR amyloidosis

Trial and drug	Population	Patients’ characteristics	Inclusion criteria	Follow up (mo)	Outcome measure	Adverse events
ATTR-ACT Tafamidis (PO daily)	264 drug 177 placebo	Median age 75 y ATTRwt (76%) ATTRv (24%) White patients (80%) Black patients (14%)	NYHA I–III History of HF NT-proBNP ≥600 6MWT >100 m	30	↓ 30% all-cause mortality and CV-related hospitalizations	None reported
APOLLO Patisiran (IV every 3 wk)	148 drug 77 placebo	Median age 62 y ATTRv (100%) White patients (72%) Asian patients (23%) Black patients (2%)	NYHA I–II ↑LVWT	18	↑ PN score ↑ QOL	Peripheral oedema (30%) Infusion reaction (20%)
NEURO-TTR Inotersen (SC every week)	112 drug 60 placebo	Median age 59 y ATTRv (100%) White patients (92%) Black patients (2%)	NYHA I–II LVWT ≥13 mm	15	↑ PN score ↑ QOL	Glomerulonephritis (3%), low platelet count (2%) Monitoring vitamin A serum levels
Berk *et al*.^69^ Diflunisal	64 drug 66 placebo	Median age 60 y ATTRv (100%) White patients (79%) Asian patients (11%) Black patients (5%)	NYHA I–III	24	↑ PN score ↑ QOL	4 drug-related discontinuations (GI bleeding, HF, glaucoma, nausea)

6MWT, 6-minute walk test; APOLLO, The Study of an Investigational Drug, Patisiran, for the Treatment of Transthyretin-Mediated Amyloidosis; ATTR-ACT, Safety and Efficacy of Tafamidis in Patients With Transthyretin Cardiomyopathy; ATTRv, variant transthyretin amyloidosis; ATTRwt, wild- type transthyretin amyloidosis; CV, cardiovascular; GI, gastrointestinal; HF, heart failure; IV, intravenous; LVWT, left ventricular wall thickness; NEURO-TTR, Efficacy and Safety of Inotersen in Familial Amyloid Polyneuropathy trial; NT-proBNP, n-terminal pro-B-type natriuretic peptide; NYHA, New York Heart Association; PN score, peripheral neuropathy score as measured by the modified neuropathy impairment score plus 7 nerve tests; PO, oral; QOL, quality of life; SC, subcutaneous.

### 9.1 TTR stabilizer

#### 9.1.1 Tafamidis

This small molecule stabilizes the circulating TTR tetramer and prevents dissociation in monomers by binding the T4-binding sites. In a phase 3 multi-centre trial, tafamidis reduced by 30% all-cause mortality and cardiovascular hospitalization in patients with HF due to ATTRwt and ATTRv-CA when compared to placebo.^63^ This was reflected in a lower rate of decline of SV after 30 months with tafamidis treatment compared with placebo. The drug can be orally administered and was safe and well tolerated. Notably, patients in NYHA class IV and estimated glomerular filtration rate < 25 ml/min/m^2^ were excluded from the study population and the consistent mortality benefit was evident only after 18–20 months of therapy, predominantly in patients in NYHA I-II.^63^ Therefore, careful selection of candidates is required as some patients with poor life-expectancy might derive no survival advantage from this treatment. The reduced benefit with tafamidis in trial patients with NYHA functional class III was postulated to be related to higher hospitalization rates in this subgroup, potentially because of a longer survival during a more severe phase of the disease.^4,9^

Tafamidis has become the first disease-modifying drug to be approved for the treatment of ATTRwt and ATTRv-CA. All other drugs are only licensed for the treatment of polyneuropathy caused by ATTR amyloidosis.

#### 9.1.2 Inhibitors of TTR gene expression

TTR is a carrier protein of retinol and vitamin A supplementation is recommended in patients receiving this class of drugs.^9^

#### 9.1.3 Patisiran

Patisiran is a small interfering RNA (siRNA) encapsulated in lipid nanoparticles that blocks the expression of TTR (both wild-type and variant TTR) in the hepatocytes by disrupting the TTR mRNA, hence reducing its hepatic synthesis. In the phase 3 multi-centre APOLLO trial^64^ on patients with polyneuropathy caused by ATTRv amyloidosis, patisiran administered intravenously at the dose of 0.3 mg/kg once every 3 weeks for 18 months significantly improved neurological status. Echocardiography was consistent with ATTRv-CA in 56% of patients enrolled in the study. In this subgroup, patisiran promoted favourable myocardial remodelling, by reducing mean LV wall thickness, relative wall thickness and serum NT-proBNP levels. In a recent CMR study, patients receiving patisiran had evidence of cardiac amyloid regression in ≈ 40% of cases (see ‘Staging and Clinical Course’ section).

#### 9.1.4 Revusiran

Revusiran is a siRNA that was investigated in a phase 3 trial enrolling patients with ATTRv-CA. The study was discontinued because of ‘an imbalance in mortality observed between patients treated with revusiran and placebo’ (https://clinicaltrials.gov/ct2/show/NCT02319005?term=revusiran) although subsequent evidence suggested an imbalance in disease severity at enrolment.

#### 9.1.5 Inotersen

Inotersen is an antisense oligonucleotide (ASO) inhibiting the hepatic production of TTR (both wild-type and variant TTR). In the phase 3 NEURO-TTR trial,^65^ inotersen administered subcutaneously at the dose of 300 mg once a week for 12 months slowed the decline in neurological manifestations and improved quality of life in patients with polyneuropathy caused by ATTRv amyloidosis. ATTRv-CA was diagnosed in 68% of the study population. No significant change in LVEF, GLS, LV wall thickness, LV mass, or E/e′ ratio was observed, but the study was not powered to investigate the impact of inotersen on ATTRv-CA.

Glomerulonephritis and severe thrombocytopenia (platelet counts <25 × 10^3^/µL) were reported in 3% of patients receiving inotersen and one patient died from intracranial haemorrhage related to thrombocytopenia. In the open-label extension with enhanced monitoring, the rate of thrombocytopenia was similar among inotersen group and placebo group and there were no cases of severe thrombocytopenia or acute glomerulonephritis. Nevertheless, patients on inotersen require regular monitoring.^9^

In a single-centre study on patients with ATTRwt and ATTRv-CA, during a follow up of 24 months, inotersen improved mean LV mass measured by CMR (−8.4%) and exercise tolerance (+ 20 m during 6MWT).^66^

## 10. Future directions

### 10.1 Advances in mechanisms of amyloid formation

Physiological fibrinolysis has been postulated to play a critical role in TTR amyloid formation in vivo. Plasmin was reported to selectively cleave TTR between residues 48 and 49 under physiological conditions in vitro leading to release of truncated and full-length oligomers that rapidly aggregate and form fibrils.^67^ Furthermore, amyloidogenic cleavage has been recently proposed as alternate mechanism to tetramer dissociation in ATTR amyloidosis. Ligands able to stabilize the circulating TTR tetramer such as thyroxine and RBP have been reported not to prevent amyloidogenic cleavage in Ser52Pro TTR.^12^ This observation supported the hypothesis that the native tetrameric TTR variant is the substrate for proteolytic cleavage in vivo and that proteolysis might be potentially a prefibrillar event in a general pathway of TTR amyloidogenesis dictated by the formation of the residue 49–127 polypeptide.^12^

These findings represent a further step for elucidating the mechanisms of TTR amyloidosis and offers insight into novel potential targets for therapy.

### 10.2 Second-generation pharmacological agents

Second-generation drugs are conjugated to N-acetyl galactosamine and have a high affinity for the asialoglycoprotein receptor on hepatocytes, thus resulting in enhanced liver uptake compared to first-generation agents.^9^

#### 10.2.1 Vutrisiran

Vutrisiran is a second-generation siRNA developed from patisiran that is administered subcutaneously every 3 months that reduced circulating TTR levels by 83% after 6 weeks of treatment.^9^ This agent has shown non-inferiority compared to patisiran in a phase 3 clinical trial of patients with ATTRv-PN (NCT04153149 and NCT03759379).

#### 10.2.2 ION-682884

ION-682884 is a second-generator ASO developed from inotersen, with which it shares the same base sequence, which is administered subcutaneously without the concerns of the adverse effects of its predecessor.^9^ This agent is currently under evaluation in a phase 3 trial enrolling patients with ATTR-CA that will investigate the potential benefit of therapy on cardiovascular mortality and recurrent cardiovascular clinical events (NCT04136171).

#### 10.2.3 Acoramidis

Acoramidis is a next generation TTR stabilizer that mimics the structural stabilizing properties of the TTR variant p.T139M. The affinity and potency of acoramidis appears to exceed that of tafamidis resulting in a more effective stabilization of circulating TTR, both wild-type and variant.^9^ Acoramidis is currently being evaluated in phase III clinical trials in patients with ATTR-CA (NCT03860935).

### 10.3 Gene editing treatments

#### 10.3.1 NTLA-2001

NTLA-2001 is a new Clustered regularly interspaced short palindromic repeats and associated Cas9 endonuclease system (CRISPR-Cas9)–based in vivo gene-editing therapy, administered by intravenous infusion, which edits TTR in the hepatocyte. This agent was recently demonstrated to decrease serum TTR protein concentrations (> 95% reduction) through targeted knockout of TTR with a dose-dependent effect in six patients with ATTRv amyloidosis with polyneuropathy. Only mild adverse events were observed in this early phase study.^68^

### 10.4 Clearance of amyloid deposits

#### 10.4.1 PRX004

PRX004 is a humanized IgG1 monoclonal antibody that binds to epitope exposed on abnormal TTR protein and is intended to promote active clearance of ATTR amyloid thought a phagocytic uptake.^9^ Although promising, the phase 1, open-label study (NCT03336580) investigating the effects of this agent in patients with ATTRv amyloidosis was prematurely terminated in 2020 because of the impact of COVID-19 pandemic.

## 11. Conclusions and areas of future investigation

ATTR amyloidosis represents a fascinating and challenging disease with many grey areas to address, from mechanisms of amyloidogenesis, to early recognition, to prognostic stratification, and to monitoring of disease course and response to treatment (Graphical Abstract). A systematic approach to the evaluation of the patient’s clinical history, clinical signs and symptoms, and awareness of key ‘red flags’ of infiltrative disease are required to increase the chance of early diagnosis. ATTR-CA is increasingly recognized following landmark advances in echocardiography, CMR, nuclear imaging and genetics, with a rapidly changing diagnostic and therapeutic landscape. Non-invasive imaging modalities are paramount in characterizing myocardial involvment in ATTR amyloidosis and will be crucial in the near future for assessing response to disease-modifying treatments, guiding clinicians on when to escalate the dose, switch or combine different therapies, and when to stop treatment in non-responders.

**Figure 6:**
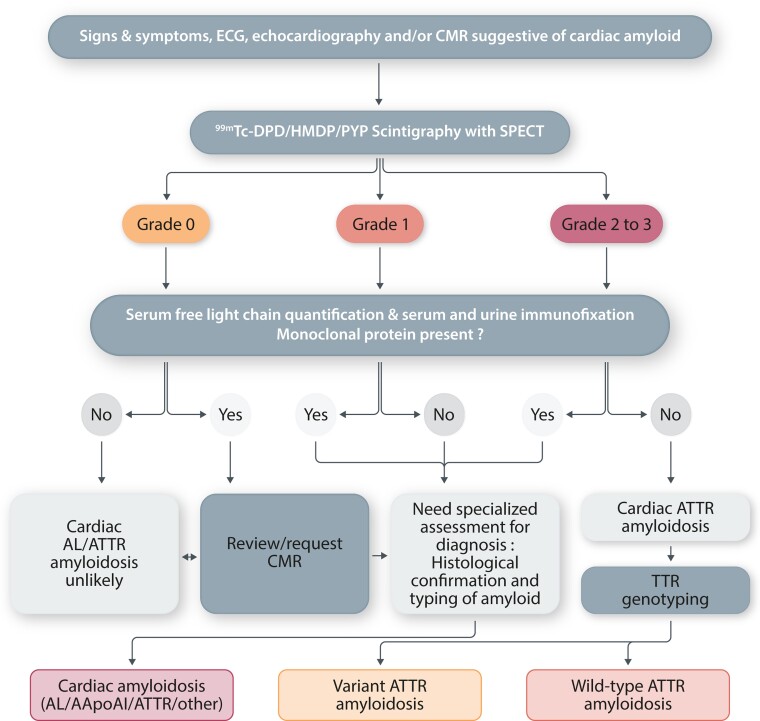
Diagnostic algorithm for patients with suspected cardiac amyloidosis. 99mTc-DPD, 99mTc-3,3-diphosphono-1,2-propanodicarboxylic acid; 99mTc- HMDP, 99mTc-hydroxymethylene diphosphonate; 99mTc-PYP, 99mTc-pyrophosphate; AApoA1, apolipoprotein A-I; AL, light chain; CMR, cardiac magnetic resonance; SPECT, single-photon emission tomography imaging; TTR, transthyretin. Readapted with permission from Gillmore *et al.*^8^.

ATTR-CA is projected to exponentially increase in the future due to improved awareness of disease in medical communities, the possibility of non-invasive diagnosis and the prolongation of the average lifespan. Addressing grey areas in clinical research will be paramount for patients’ management:

- Characterizing further biological mechanism of the ‘amyloidogenic cascade’;
- Differentiating indolent ‘cardiac accumulation’ associated with aging from ATTR-CA, the ‘authentic’ infiltrative disease;
- Understanding the epidemiology of ATTR amyloidosis in the various clinical settings;
- Maximizing the application of SPECT imaging for diagnostic and prognostic purposes;
- Exploring the role of PET imaging with amyloid tracers for differentiating between AL and ATTR amyloidosis and monitoring disease course;
- Defining the minimal disease threshold to justify the initiation of novel treatments;
- Defining the criteria to identify patients with advanced ATTR-CA in whom significant benefit from initiation of disease-modifying drugs is unlikely.

A wide horizon of possibilities is unfolding in ATTR amyloidosis and awaits discovery.

## Data Availability

No new data were generated or analysed in support of this research.
